# Supplementary figures and images for: DUSP4 protects BRAF- and NRAS-mutant melanoma from oncogene overdose through modulation of MITF
Source: Life Sci Alliance. 2022 May 17;5(9):e202101235. doi: 10.26508/lsa.202101235 (PMC9113946; doi:10.26508/lsa.202101235)

SOURCE DATA

Figure 4A

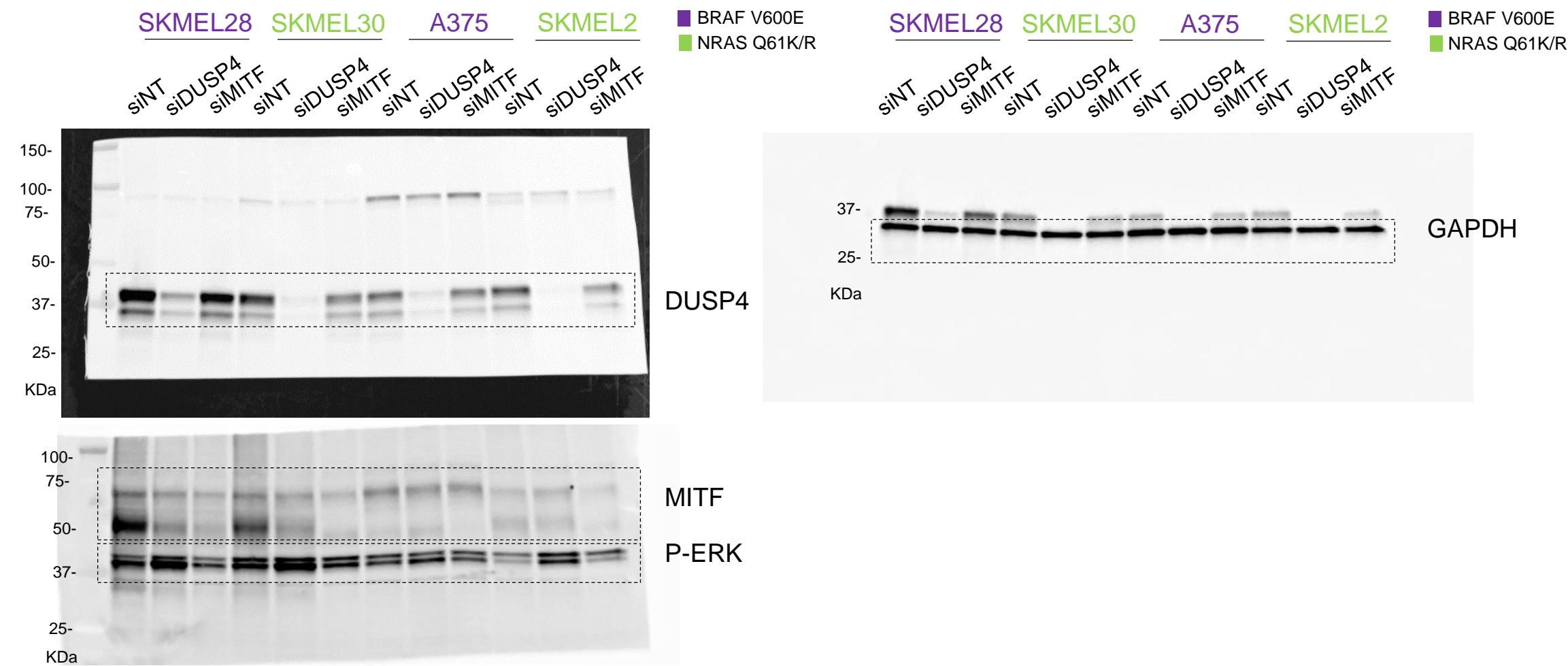

## SOURCE DATA

### Supplementary Figure 5A

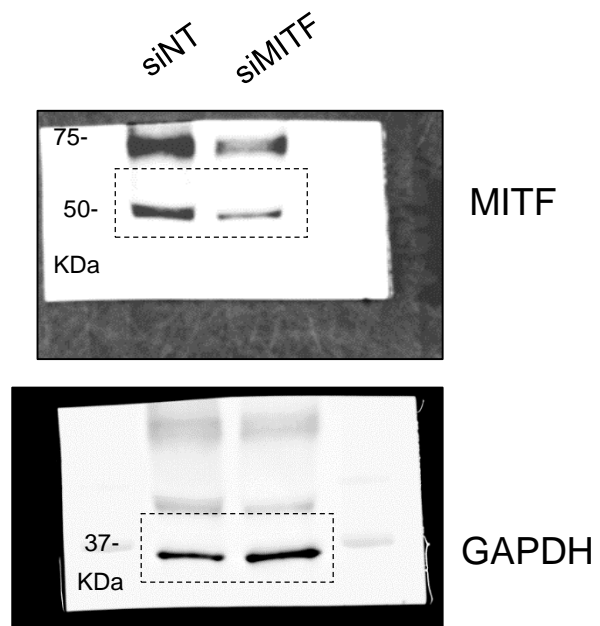

Supplement: Supplementary file 3 [file LSA-2021-01235_SdataF4_FS5.pdf]
