# Supplementary material for: DUSP4 protects BRAF- and NRAS-mutant melanoma from oncogene overdose through modulation of MITF
Source: Life Sci Alliance. 2022 May 17;5(9):e202101235. doi: 10.26508/lsa.202101235 (PMC9113946; doi:10.26508/lsa.202101235)
Supplement: Supplementary file 4 [file LSA-2021-01235_TableS3.docx]

SUPPLEMENTARY MATERIAL

| Cell Lines | Disease Stage | BRAF | NRAS | Other mutations | Age/Gender | Pathology | Site |
| --- | --- | --- | --- | --- | --- | --- | --- |
| **WM3623** | Metastasis | WT | Q61K | ARID2 | 48/Male | Right Lymph Node Resection, Level 5: Metastatic Malignant Melanoma | Right Neck Lymph Nodes |
| **WM1366** | VGP | WT | Q61L | DDX3X | 79/Male | SSM, Stage IV tumor | Right Forearm |

Supplementary Table 3. Melanoma cell lines depicting NRAS Mutations. Patient derived tumor cells are classified according on the stage of disease, mutations, age, gender, pathology and site of origin. Abbreviations: Vertical Growth Phase (VGP), Superficial Spreading Melanoma (SSM).
