# Supplementary material for: DUSP4 protects BRAF- and NRAS-mutant melanoma from oncogene overdose through modulation of MITF
Source: Life Sci Alliance. 2022 May 17;5(9):e202101235. doi: 10.26508/lsa.202101235 (PMC9113946; doi:10.26508/lsa.202101235)
Supplement: Supplementary file 5 [file LSA-2021-01235_TableS4.docx]

SUPPLEMENTARY MATERIAL

| Pool Catalog Number | Gene Symbol | Gene Accession |
| --- | --- | --- |
| L-003484-02 | DUSP1 | NM_004417 |
| L-003565-00 | DUSP2 | NM_004418 |
| L-003963-00 | DUSP4 | NM_001394 |
| L-003566-00 | DUSP5 | NM_004419 |
| L-003964-00 | DUSP6 | NM_022652 |
| L-003567-00 | DUSP7 | NM_001947 |
| L-003568-00 | DUSP8 | NM_004420 |
| L-003569-00 | DUSP9 | NM_001395 |
| L-003965-00 | DUSP10 | NM_144729 |
| L-007888-00 | DUSP14 | NM_007026 |
| L-007890-00 | DUSP16 | NM_030640 |
| L-007890-00 | DUSP26 | NM_024025 |
| L-007890-00 | PPP2R1A | NM_014225 |
| L-007890-00 | PPP2R1B | NM_181699 |
| L-008027-00 | PPP2R2A | NM_002717 |
| L-008027-00 | PPP2R2B | NM_181676 |
| L-008027-00 | PPP2R2C | NM_181876 |
| L-008027-00 | PPP2R2D | NM_001003656 |
| L-010259-00 | PPP2R3A | NM_181897 |
| L-010259-00 | PPP2R3B | NM_199326 |
| L-010259-00 | PPP2R3C | NM_017917 |
| L-010259-00 | PPP2R5A | NM_006243 |
| L-017592-00 | PPP2R5B | NM_006244 |
| L-017592-00 | PPP2R5C | NM_178588 |
| L-017592-00 | PPP2R5D | NM_180977 |
| L-003022-00 | PPP2R5E | NM_006246 |
| L-003022-00 | PPP2CA | NM_002715 |
| L-032298-00 | PPP2CB | NM_004156 |
| L-032298-00 | PTEN | NM_000314 |
| L-019459-00 | RNF125 | NM_017831 |
| L-019459-00 | NF1 | NM_000267 |
| L-009366-00 | USP28 | NM_020886 |
| L-009366-00 | SPRY1 | NM_199327 |
| L-009799-00 | SPRY2 | NM_005842 |
| L-009799-00 | SPRY4 | NM_030964 |
| L-003598-01 | SPRED2 | NM_181784 |
| L-003598-01 | STAG2 | NM_006603 |
| L-003023-00 | STAG3 | NM_012447 |
| L-003023-00 | PTPN18 | NM_014369 |
| L-003916-00 | PTPN11 | NM_002834 |
| L-003916-00 | PTPN7 | NM_080589 |
| L-006076-00 | PEA15 | NM_003768 |
| L-006076-00 | PTPRR | NM_130846 |
| L-027339-00 | ON-TARGETplus Non-targeting Control |  |
| L-005206-00 | ON-TARGETplus Non-targeting Control |  |
| L-015457-01 | ON-TARGETplus Non-targeting Control |  |
| L-018590-00 | ON-TARGETplus Non-targeting Control |  |

Supplementary Table 4. List of siRNAs included in the siRNA-cherry pick library.
